# 1st Conference of the South African Society of Biomechanics 28–29 October 2021: “FOUNDATIONS TO FRONTIERS”

**DOI:** 10.17159/2078-516X/2022/v34i1a13377

**Published:** 2022-01-01

**Authors:** Helen Bayne, Yumna Albertus, John Cockcroft, Mark Kramer

**Affiliations:** University of Pretoria; University of Cape Town; Stellenbosch University; North West University

The South African Society of Biomechanics (SASB) is a voluntary association that was established in 2021 to support the growth of biomechanics in the country. The mission of SASB is to advance the field of biomechanics in South Africa by supporting high quality research and promoting the translation of research into practice by (i) providing a forum for the exchange of knowledge on biomechanical theory and application, (ii) supporting the training and education of student biomechanists, and (iii) facilitating networking between practitioners, researchers, institutions and industry.

The 1^st^ Conference of SASB was held as a virtual event on 28–29 October 2021. The theme of the conference was “Foundations to Frontiers” and the world class line-up and keynote and tutorial speakers (listed below) delivered presentations that addressed the full continuum from fundamental biomechanical methods to ground breaking research and innovation.

- Jacqueline Alderson: “Poses, Loads and Bridges: The Asset of Rigour”
- Felipe Carpes. “Why are cross-bridges important in biomechanics? The benefit of being interdisciplinary”
- Ezio Preatoni. “Skills, coordination and movement variability in sport: potential and pitfalls”
- Amy Wu. “Towards dynamic locomotion and balance at the intersection of biomechanics and robotics”
- John Cockcroft. “Foundations for building a data processing pipeline: a practical introduction to typical tasks and available tools”
- Friedl de Groote. “Musculoskeletal modelling and simulations to analyze measured data and predict movement patterns: overview and hands-on demo in OpenSim”

Attendees were also treated to a conversational session with Erica Bell about lessons learned during her journey in biomechanics, and talks from the conference sponsors – “Motion capture: The paradox of choice”, by Felix Tsui (Vicon Motion Systems Ltd) and “Is markerless tracking of 3D human pose accurate”, by Scott Selbie (Theia Markerless, Inc).

Awards were presented for the top three scientific presentations to Charné Britz, Devon Coetzee and Cassidy de França. Their abstracts are published in these proceedings.

The recordings of selected sessions are publicly available on the SASB Vimeo Channel and all keynote and tutorial sessions are available to SASB members on our website: www.biomechsa.org.

**Organisers:** Helen Bayne, *University of Pretoria*; Yumna Albertus, *University of Cape Town;* John Cockcroft, *Stellenbosch University*; Mark Kramer, *North West University*

