# Supplementary material for: 1st Conference of the South African Society of Biomechanics 28–29 October 2021: “FOUNDATIONS TO FRONTIERS”
Source: S Afr J Sports Med. 2022 Jan 1;34(1):v34i1a13377. doi: 10.17159/2078-516X/2022/v34i1a13377 (PMC9924609; doi:10.17159/2078-516X/2022/v34i1a13377)
Supplement: Related texts

# Abstract: The effect of eccentric and concentric cycling ergometry rehabilitation on gait, post total knee arthroplasty

**Published:** 2022-01-01

**Authors:** Yumna Albertus, Sandhya Silal, Michael Posthumus, Nicholas Tam

**Affiliations:** 1 Health through Physical Activity, Lifestyle and Sport Research Centre (HPALS), Department of Human Biology, University of Cape Town. International Federation of Sports Medicine (FIMS) Collaborative Centre of Sports Medicine, Cape Town, South Africa

## Introduction

Total knee arthroplasty (TKA) procedures are known to improve joint-specific outcomes. However, functional deficits post-surgery have been noted, particularly in terms of movement abnormalities and quadriceps weakness. Eccentrically-based rehabilitation has been shown to improve muscle strength and reduce metabolic demand. Thus we aimed to determine the effects of an eight-week eccentric and concentric cycling ergometry exercise intervention in TKA recipients, especially on the joint kinematics and muscle activity during walking gait.

## Methods

Eighteen participants, three to nine months post total knee arthroplasty, were recruited and randomly assigned to either an eccentric (ECC) or concentric (CON) cycling exercise intervention group. Participants performed three exercise sessions weekly over a progressive eight-week period on the Grucox Isokinetic Ergometer. Walking gait biomechanics, knee functional outcomes and quality of life were recorded pre- and post-intervention.

## Results

The CON group knee flexion range of movement and peak knee flexion increased significantly during the swing phase of gait (p = 0.021) post-intervention. No significant changes in kinematics and kinetics were noted in the ECC group post-intervention. The ECC intervention group showed neuromuscular changes with a decrease in biceps femoris activity during the load acceptance phase of gait (ECC Pre 12.1% (11.4% – 15.1%) and Post 8.1% (5.9% – 12.7%); p = 0.021). Significant correlations between knee joint stiffness and the quadriceps:hamstring co-activation ratios were observed in the CON group pre-intervention (r = −0.68; p = 0.042).

## Conclusion

The eccentric intervention resulted in neuromuscular adaptations consistent with a move towards a more typical asymptomatic gait pattern. The concentric intervention yielded kinematic changes however, these results show eccentric training’s role in early stage post-operative rehabilitation is limited. Based on the findings from this exploratory study, the benefit of eccentric training as an adjunct to rehabilitation and its role in contributing to greater improvements in the restoration of functional ability post-TKA needs to be further explored.

# Abstract: Characterizing the influence of a prosthesis on the shot put movement

**Published:** 2022-01-01

**Authors:** Nathanael Boulle, Taahirah Mangera, Frank Kienhöfer

**Affiliations:** 1 School of Mechanical, Industrial and Aeronautical Engineering, University of Witwatersrand

## Introduction

Competitive able-bodied athletes have shown to benefit from biomechanical analysis. However, there is a paucity of research into the movement science of disabled athletes and uncertainty as to whether the biomechanical principles of able-bodied athletes can be applied to disabled athletes. This investigation seeks to provide principles for improved shot put outcomes for an athlete using a lower limb prosthesis, and determine whether able-bodied literature can be applied to disabled movement science for the shot put movement.

## Methods

Motion data was collected using an Xsens MVN Analyse motion capture system and ground reaction force data was collected using a FDM pressure walkway. The captured data was used to articulate and validate a rigid multibody model developed in Simscape Multibody, a simulation environment provided by MATLAB. The model was lower body specific and computed results including combined segmental velocity data, joint torques, normal and frictional forces. The simulation was validated using measured displacements from Xsens as well as measured ground reaction forces from the pressure walkway compared against computed normal force data.

## Results

The main biomechanical principles of able-bodied shot put athletes were found to be concerned primarily with segmental velocity, centre of gravity (CG) profile, feet sequencing and trunk. The principle investigation emphasized the initial glide in the preamble as well as the delivery stride. Significant areas of influence for the prosthesis are characterized by a reduced delivery stride width, impaired sequential muscle activation and difficulty in providing an effective base from which the upper body segments can extend

## Conclusions

The evidence of the investigation indicates that the utilization of able-bodied movement principles is useful in disabled sports science. Moreover, the principles could even be observed as fundamental theory for any given shot putter, or by extension any projectile based sport.

# Abstract: Reliability of step impact asymmetry metrics obtained using wearable sensors during netball-specific drills

**Published:** 2022-01-01

**Authors:** Charné Britz, Helen Bayne, James Clark

**Affiliations:** 1 Department of Physiology and Sport, Exercise Medicine and Lifestyle Institute, Faculty of Health Sciences, University of Pretoria

## Introduction

Inter-limb asymmetry in lower limb biomechanical load associated with step impacts has been proposed as a measure that may be useful for monitoring injury risk and rehabilitation progression. Inertial measurement units (IMUs) are feasible monitoring tools to monitoring cumulative step impact load for each limb separately. Studies show that IMUs can reliably assess step impacts during running-based sport movements. Little is known about the reliability of inter-limb asymmetries derived from step impact metrics during netball-specific movements. The aim of this study was to determine between-day reliability of impact load asymmetry in netball-specific drills.

## Methods

Twenty-two healthy collegiate female netball players (age: 20 ± 2 years, height: 177.6 ± 7.0 cm, mass: 69.9 ± 8.3 kg) volunteered for a study approved by the institution’s ethics committee and performed six standardised netball-specific tasks on two test occasions separated by one day. Impact load was obtained for both limbs using an IMU (IMeasureU Blue Trident, Vicon Motion Systems Ltd) affixed to the lower tibia. Inter-limb impact load asymmetry was calculated: ((Right leg − Left leg)/(Right leg + Left leg) × 100) for each task and compared between the two testing occasions to determine between-day reliability using the coefficient of variation (CV) and intraclass correlation coefficients (ICC).

## Results

Impact load for each limb had acceptable reliability on most tasks (CV range: 11.1–19%, ICC range: 0.52 – 0.78), except for a small-sided game (Left: CV: 23%, ICC: 0.44; Right: CV: 21%, ICC: 0.38). Impact load asymmetry showed very poor reliability across all tasks (CV: 83.4 – 731% ICC: −0.04 – 0.17).

## Conclusion

Impact load asymmetry was not reliable between sessions for netball-specific drills, although the measure for each limb had acceptable reliability. Practitioners should be aware of the limitations of this asymmetry metric.

# Abstract: Linking clinical outcomes of injury and running biomechanics to kinetic risk factors for injury: how novice runners respond to running in footwear with reduced cushioning

**Published:** 2022-01-01

**Authors:** Devon Ross Coetzee, Ross Tucker, Nicholas Tam, Yumna Albertus

**Affiliations:** 1 Health through Physical Activity, Lifestyle and Sport Research Centre (HPALS), Department of Human Biology, University of Cape Town. International Federation of Sports Medicine (FIMS) Collaborative Centre of Sports Medicine, Cape Town, South Africa; 2 Department of Organizational Psychology, School of Management Studies, University of Cape Town, South Africa

## Introduction

The high prevalence of running injury has prompted the need to understand the link between biomechanics and injury outcome. This study aimed to investigate the potential link between running biomechanics and clinical measures of injury, namely pain or discomfort and bone oedema. Additionally, this study aimed to determine the intuitive kinetic and kinematic adaptations during a 12-week running intervention when running in footwear with reduced cushioning, with focus on any changes that may influence injury risk.

## Methods

Fifty-four novice runners were assigned to the traditionally cushioned (TC; n = 32) or the reduced cushioning group (RC; n = 22). Bone oedema of the lower leg (MRI) and pain or discomfort was measured. Baseline and post-intervention biomechanical testing included motion capture and force plates to assess over ground running at 3.0 m.s^−1^. Key variables assessed included sagittal lower limb kinematics, vertical ground reaction force (vGRF) and initial loading rate (ILR).

## Results

Bone oedema was associated with greater ILR, however no other relationships existed between other injury outcomes such as pain or discomfort and injury itself. Only one participant (RC) transitioned from a rearfoot to a forefoot strike pattern throughout the intervention. The RC group increased knee flexion angle over time (baseline of 16.1º ± 4.5 to post-intervention of 19.7º ± 3.0; p < 0.01) and were 3.9 times more likely to reduce foot strike angle when compared to the TC group. RC participants who reduced foot strike angle presented with a significant reduction in vGRF and accumulative load.

## Conclusion

Footwear with reduced cushioning may result in kinematic strategies to dampen loading rates that would normally be dissipated by the midsole of the shoe. This may have important implications for risk of injury as ILR may be associated with an increased risk of developing bone oedema of the lower leg.

# Abstract: Countermovement jump performance and asymmetry after lower limb injury

**Published:** 2022-01-01

**Authors:** Cassidy de França, Helen Bayne

**Affiliations:** 1 Department of Physiology and Sport, Exercise Medicine and Lifestyle Institute, Faculty of Health Sciences, University of Pretoria

## Introduction

Due to the high incidence, burden and severity of lower limb injuries, this study aimed to identify differences in performance and interlimb asymmetry during the countermovement jump (CMJ) between uninjured and previously injured athletes post lower limb injury.

## Methods

Previously injured (INJ: n = 12) and uninjured athletes (CON: n = 16) completed self-reported injury history questionnaires and routine CMJ testing (INJ 3.9 ± 1.8 months post injury). Vertical ground reaction force was captured using dual force plates and force-time data was used to analyse between-group differences for CMJ phase-specific asymmetry and performance variables. Ethical approval was obtained prior to testing. Groups were compared using independent samples t-tests (Student’s T, alternatively the Mann-Whitney U) and Cohen’s d effect sizes.

## Results

Groups showed no differences and small effect sizes for jump height, modified reactive strength index, peak power, eccentric deceleration impulse, peak landing force, and force at zero velocity, as well as asymmetry in concentric impulse, eccentric deceleration impulse, and take-off peak force asymmetry. Compared to controls, previously injured athletes produced greater asymmetry in peak landing force (INJ: 9.8 ± 4.9%, CON: 6.0 ± 4.5%, p = 0.04) (d = 0.83), which is a novel finding. Injured athletes produced lower force at zero velocity (*d* = 0.73), peak power and greater concentric impulse (*d* = 0.70). Despite returning to sport, significant interlimb asymmetries in the landing phase of the CMJ exist up to 6 months post injury, although jump performance has been restored. This study is limited by the small sample size and used of self-reported retrospective injury data, but the preliminary analysis will be used to inform future studies.

## Conclusion

Despite normal CMJ jump performance post injury, compensatory movement strategies and asymmetries may still exist up to 6 months post injury. This may be related to an increased risk of a non-contact lower limb injury in future.

# Abstract: Step-to-step changes in foot-shank coordination during initial sprint acceleration

**Published:** 2022-01-01

**Authors:** Byron J. Donaldson, Neil E. Bezodis, Helen Bayne

**Affiliations:** 1 Department of Physiology, Faculty of Health Sciences, University of Pretoria; 2 A-STEM Research Centre, Swansea University

## Introduction

Initial acceleration is characterised by step-to-step changes in kinematics. The first steps see changes in the shank angle and changes in the foot position and orientation. Coaches focus on ankle mechanics during acceleration, which is directly influenced by the motion of these two segments. Understanding the coordination between shank and the foot during this phase can improve understanding of acceleration technique and the contribution of shank and foot to ankle motion.

## Methods

Twenty-one sprinters (15 male, 100 m PB 10.47 ± 0.42 s; 6 female, 100 m PB 11.70 ± 0.24 s) provided informed consent and the study was approved by the institutional ethical review committee. Sprinters performed three ≥20 m starts from blocks and the fastest trial was analysed. Sagittal plane kinematics were captured using inertial measurement units (200 Hz; MyoMotion, Noraxon, USA). Individual and group mean foot-shank coordination over the first four steps was determined using vector coding techniques. Step-to-step changes were assessed based on the coordination bin frequencies and coupling angle difference scores (CA_Diff_) between adjacent steps.

## Results

Step-to-step coordination differences were largest between step 1 and 2 (CA_Diff_ = 29.7 ± 11.3%), with progressively smaller differences between steps 2–3 (CA_Diff_ = 23.6 ± 6.9%) and 3–4 (CA_Diff_ = 16.9 ± 7.2%). There was greater anti-phase coordination (AP foot (−) 19%, AP foot (+) 16%) during the first step, however became dominated by in-phase shank (−) (IPS-) and foot (−) (IPF-) coordination over step 2, 3 and 4 (IPS- 45, 44, 42%, IPF- 18, 16, 18% respectively).

## Conclusion

Sprinters utilise potentially distinct foot-shank coordination during the first step. There are large step-to-step coordination changes that get progressively smaller over the first four steps of a sprint. Coordination is mostly in phase and foot dominant, highlighting an important role of the foot in rotation and ankle motion.

# Abstract: Physiological and sprint-kinetics related to YYIR1 performances in soccer players

**Published:** 2022-01-01

**Authors:** Mark Kramer, Martinique Sparks, Ben Coetzee

**Affiliations:** 1 Physical Activity, Sport, and Recreation (PhaSRec) Research Focus Area, North-West University

## Introduction

Although high-speed running ability and cardiorespiratory fitness (CRF) are touted as associative predictors of the Yo-Yo intermittent recovery run (YYIR1) test performance, the evidence substantiating this is inadequate. We therefore evaluated direct CRF via portable spirometry during the YYIR1, and the anaerobic speed reserve (ASR) by modelling the sprint-kinetics of a 40-m all-out maximal sprint speed (MSS) test.

## Methods

Twenty-three male soccer players were recruited for the study. After informed consent was provided, each participant completed a YYIR1 and MSS test. Heart rate and pulmonary oxygen uptake were continuously recorded during the YYIR1. Sprint-kinetics were obtained and modelled from a 40-m all-out sprint test using photocells placed at 0-m, 5-m 10-m, 20-m, 30-m and 40-m distances.

## Results

The data revealed significant differences between observed and predicted V̇O_2_max values (p < 0.001). The Bland-Altman analysis showed a mean bias between observed and predicted V̇O_2_max of 31%, with the limits of agreement spanning 16% above and below the mean. The ASR, which served as a marker of high-speed running capacity, showed statistically significant correlations with the following sprint-based parameters: max speed (r = 0.86, p <.001), absolute power-asymptote (r = 0.65, p < .001), and relative power-asymptote (r = 0.68, p < .001), but not with any YYIR1 parameters (all r < 0.20, p > .469).

## Conclusions

The intermittent nature of the YYIR1 provides a unique challenge that is not captured by data derived from either physiological- or sprint-kinetic assessments. It is unlikely that YYIR1 performances are dependent on, or predicted by, high-speed running ability, at least in university-level soccer players. Subsequently, we do not recommend the use of YYIR1 for determining CRF, and would suggest that alternative tests with greater validity, reliability, and physiological utility be used for such purposes.

# Abstract: Gait adaptability and biofeedback in older adults with diabetes

**Published:** 2022-01-01

**Authors:** Suzanne Martin, Simon Taylor, Rajna Ogrin, Rezaul Begg

**Affiliations:** 1 Institute for Health and Sport, Victoria University, Melbourne, Victoria, Australia; 2 Bolton Clark Research Institute, Melbourne, Victoria, Australia

## Introduction

Modulation of gait parameters for adjusting foot placement to changes in the environment is called gait adaptability. Although older adults with diabetes mellitus report fall incidents more frequently, their gait adaptability has not been investigated well. In this PhD project, the effects of diabetes, ageing and biofeedback on gait adaptability and the agreement between two presented overground and treadmill assessments in this project for future application of the developed tools were investigated.

## Methods

Participants were 16 young adults, 16 healthy older adults and 16 older adults with diabetes. They completed overground gait and gait adaptability tests with four random conditions (step shortening, step lengthening, obstacle avoiding, walking through) at a preferred speed for quantifying gait parameters and foot placement adjustments. They then completed treadmill tests with and without targeted biofeedback. Foot placement adjustments were measured and compared without biofeedback during overground and treadmill walking. Three-dimensional motion capture systems, force platforms, MATLAB and Visual3D server software quantified spatiotemporal parameters and foot placement adjustments (errors). They also presented visual targeted biofeedback. Analysis of variance (ANOVA) tested the effects of groups and conditions whereas Bland and Altman plots assessed the agreement between the overground and treadmill tests.

## Results

Gait parameters were not significantly different between groups when walking in the baseline. However, stance time, step velocity, double support time and foot placement adjustments increased in older adults with diabetes when they responded to goal-tasks in adaptability tests. All groups could use targeted biofeedback for their foot placement adjustments during online correction of their tasks. Foot placement adjustments were comparable between overground and treadmill tests.

## Conclusions

Gait adaptability was impaired in the older adults with diabetes. However, they could use targeted biofeedback in the form of visual feedback on the monitor to reduce their errors (i.e. they improved their foot placement adjustments). Novel adaptability tools in this PhD project can train more adaptable gait patterns in older adult with diabetes.

# Abstract: Development of intelligent wearables for the estimation of motion kinematics and kinetics

**Published:** 2022-01-01

**Authors:** Marion Mundt

**Affiliations:** 1 Institute of Biomechanics and Orthopaedics, German Sport University Cologne, Cologne, Germany

## Introduction

Motion analysis that provides insight into joint kinematics and kinetics is still restricted to laboratory set-ups. To overcome this, the aim of this thesis was to develop an easy-to-use and easy-to-interpret inertial-sensor-based motion analysis system leveraging artificial neural networks (ANNs). During a training process, ANNs learn to adapt their weights and biases to predict the output of unknown test samples. This thesis aimed to estimate the ground reaction force and three-dimensional angles and moments of hip, knee and ankle joint while establishing the optimum artificial neural network architectures using simulated and measured inertial sensor data.

## Methods

A framework to simulate inertial sensors’ data based on marker trajectories collected by optical systems was developed using historical datasets. The approach was validated on newly collected data of a custom IMU system. Simulated and measured data was used as input to fully-connected feedforward (FF) and recurrent long short-term memory (LSTM) neural networks.

## Results

There was a good agreement of the estimated kinematics and kinetics to the ground truth data for walking and fast changes of direction. Enlarging the dataset with augmentation techniques improved the results. Both neural networks resulted in high accuracy, with the FF network achieving greater accuracy than the LSTM.

## Conclusions

Although the FF network achieved greater accuracy than the LSTM neural network, LSTMs require less pre-processing, do not require time normalised data and are able to make real-time predictions. Therefore, LSTMs should still be considered in scenarios where these characteristics are favourable. The promising results of this thesis lay the foundations for biomechanical analysis outside of the lab, thus prompting further research in this direction.

# Abstract: Establishing structural criteria for heavy-load carriage systems

**Published:** 2022-01-01

**Authors:** Dario Schoulund

**Affiliations:** 1 Faculty of Engineering, Built Environment and Information Technology, University of Pretoria

## Introduction

Ancient and modern types of load carriage systems (LCS) coexist today worldwide. While there is a general agreement regarding which LCS are superior to others in terms of energy expenditure, the question why remains unclear. We set to answer from a structural perspective, hypothesizing that if a relationship between performance and LCS structural configuration could be established, a theoretically ideal arrangement may be defined.

## Methods

Five reportedly efficient methods of load carriage, namely tumpline, backpack, springy poles, head load and double pack were analysed as determinate structures (∑Fx, ∑Fy and ∑M = 0), with a constant load of 150 N. Excursion patterns for the load were considered at a walking speed of 1.3 m.s^−1^. The focus is on the relationship among load transfer areas, load paths, centre of masses, moments and the resultant range of forces exerted onto the user. The results were then compared against the Extra Load Index (ELI) values reported in literature.

## Results

Body centre of mass and load centre of mass horizontal eccentricity proved to be the main detrimental factor influencing gait adaptations and the development of shear forces for both static and dynamic conditions. Load paths’ geometry, axial loads, and the magnitude of shear forces combined determined the path’s efficiency. Load transfer areas and the quality of the supports requiring pre-stressing, should also be consider an external additional force acting onto the user. Finally, pressure distribution showed no correlation to structural arrangement.

## Conclusions

There exists a partial correlation between superior structures and metabolically efficient LCS. The structural analysis led to the definition of a theoretically ideal structural arrangement, which could be a promising path for further research and design. While base structural arrangements represent a critical foundation for an LCS, other less studied aspects of performance -mostly qualitative-, play a more important role than assumed for the overall performance and choice of a given LCS.

# Abstract: Acute kinetic and kinematic differences between minimalist sandal, shod and barefoot running in habitually shod male recreational trail runners

**Published:** 2022-01-01

**Authors:** Matthew Swart, Simon De Waal, Ranel Venter

**Affiliations:** 1 Department of Sport Science, Stellenbosch University

## Introduction

A plethora of studies on minimalist running shoes exists; however, none have explored the acute biomechanical effects of running in a minimalist sandal within a habitually shod population. This study aimed to investigate the acute effects of minimalist running sandals on vertical loading rates and selected lower-limb kinematics during submaximal-level treadmill running.

## Methods

Thirteen male recreational trail runners ran on a level (0º) instrumented treadmill, at three different submaximal velocities (2.22 m.s^−1^, 2.78 m.s^−1^and 3.61 m.s^−1^), using three different footwear conditions: barefoot, minimalist sandal (Xero Shoes, Colorado, US) and their conventional trail-running shoes. Supplementary to the treadmill, an inertial measurement unit system was used to capture lower-limb 3D kinematic and ground reaction force data synchronously, to measure vertical average loading rate (VALR), vertical instantaneous loading rate (VILR), foot strike angle (FSA), ankle dorsiflexion (ADA) and knee flexion angles at contact, as well as knee flexion range of motion (ROM) during the stance phase.

## Results

Pairwise comparisons revealed no significant differences between minimalist sandal and barefoot running. Differences were found in FSA (p < 0.05) and ADA (p < 0.05) at contact between minimalist and barefoot versus shod running. Moreover, increased VALR and VILR were experienced in a minimalist and barefoot condition as opposed to running in a shod condition, although the differences were not significant.

## Conclusion

Running in a minimalist sandal is similar to barefoot running. Runners seeking to change from a habitually shod condition to running in a minimalist sandal or barefoot condition, should progress with caution due to the higher acute loading rates.
